# *Galleria mellonella* as a host model to study *Candida glabrata* virulence and antifungal efficacy

**DOI:** 10.1080/21505594.2017.1347744

**Published:** 2017-08-01

**Authors:** Lauren Ames, Sarah Duxbury, Bogna Pawlowska, Hsueh-lui Ho, Ken Haynes, Steven Bates

**Affiliations:** Biosciences, College of Life and Environmental Sciences, University of Exeter, Exeter, Devon, UK

**Keywords:** *Candida glabrata*, fungal pathogenesis, *Galleria mellonella*, host model, virulence

*Candida* species are common human fungal pathogens causing a wide range of clinical diseases, ranging from superficial infections to life-threatening systemic disease. Superficial infections include vaginal candidiasis which affects over 75% of women during their lifetime with 5% of women suffering debilitating recurrent infections.^1^^,^^2^ Life-threatening systemic *Candida* disease is the fourth most common nosocomial blood stream infection, affecting those undergoing chemotherapy, recovering from surgical procedures or major burns, transplant recipients and AIDs patients. The crude mortality rate associated with these infections is high, ranging from 46–75%, and current estimates suggest at least 400,000 life-threatening infections occur annually.^1^^,^^3-6^ *Candida albicans* is the predominant cause of invasive candidiasis, although in the last 3 decades there has been a rise in the incidence of non-*albicans Candida* species with *Candida glabrata, Candida parapsilosis* and *Candida tropicalis* being the other main agents causing disease. Of these, *C. glabrata* is the second most common cause of invasive candidiasis in the USA and Central and Northern Europe, and it has been associated with higher hospital costs.^5^^,^^7-9^ The basis of this increasing incidence of *C. glabrata* is not fully understood, however, it could be partially attributed to the higher innate tolerance *C. glabrata* displays to azole antifungals alongside its greater potential to develop drug resistance coincident with therapy.^10^^,^^11^

Murine models of infection are typically viewed as the gold standard for fungal virulence studies. However, although these models allow the host-pathogen interaction to be studied *in vivo* they do come with caveats associated with cost, legislation, and careful ethical considerations. Furthermore, with the development of large scale mutant libraries alternative, more ethically acceptable, models are required to identify interesting virulence targets while limiting the use of mice.^12^^,^^13^ Given the caveats associated with murine models of infection mini-host models, mainly invertebrates, have been explored as alternative models for fungal infection. These models include amoeba (*Dictyostellium discoideum*), nematodes (*Caenorhabditis elegans*), fruit fly (*Drosophila melanogaster*) and the greater wax moth larvae (*Galleria mellonella*).^14-16^ *G. mellonella*, a lepidopteran, was first described as a mini-host for *Candida* species by Kavanagh and coworkers,^17^^,^^18^ and has received particular attention as an alternative host as it displays some important advantages. The *G. mellonella* larvae can be incubated at 37°C, allowing virulence to be studied at human body temperature, and an exact inoculum of the pathogen can be delivered by injection. Furthermore, the assays are inexpensive and simple to perform, allowing large numbers of larvae to be infected and thus increasing the statistical power of the assay. Finally some aspects of the *G. mellonella* immune response show similarities with the innate immune response of mammals.^14^^,^^15^^,^^19^ Given these advantages the model has now been developed for a wide range of fungal pathogens, including several *Candida* species.^17^^,^^18^^,^^20-22^

To study *C. glabrata* infection in mice immunosuppression is usually required, and fungal burdens and persistence are normally used as a parameter for virulence due to the absence of mortality.^23^ This, along with the recent development of large scale mutant libraries, makes alternative models for studying *C. glabrata* virulence an attractive proposition. Initial reports on establishing the *G. mellonella* model for testing the virulence of *Candida* species only reported a low level of killing of larvae by *C. glabrata*.^18^^,^^24^^,^^25^ However, we and others^26^^,^^27^ have now shown that a faster rate of killing by *C. glabrata* is seen when using a higher pathogen concentration. In this work we provide the first detailed report on the ability of *C. glabrata* to grow and cause lethal infections in *G. mellonella* in a dose dependent manner. Furthermore, we have shown that this model can be used to assess the relative virulence of *C. glabrata* clinical isolates, and that the analysis of mutant strains demonstrates an overlap with results published using murine infection models. Finally, we have shown that antifungal efficacy in the *G. mellonella* model correlates with the *in vitro* susceptibility profile of *C. glabrata*. Therefore, the *G. mellonella* model can be used to study both *C. glabrata* virulence and antifungal efficacy.

**Figure 1.**
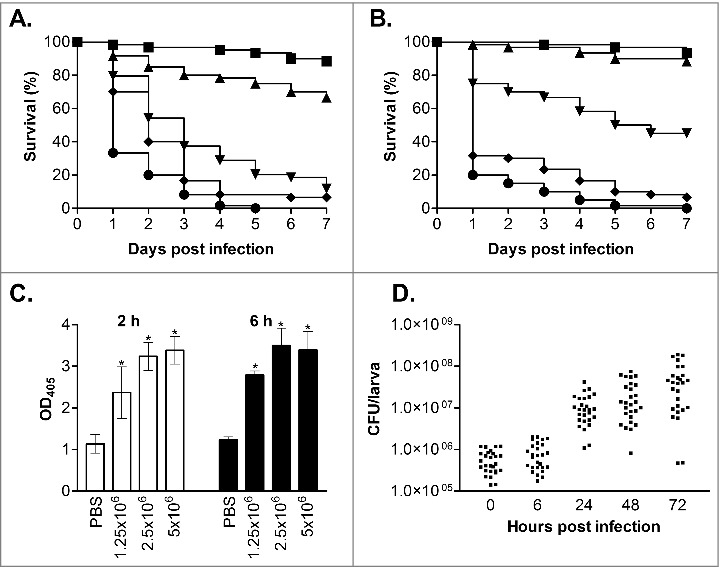
Virulence of *C. glabrata* in *G. mellonella*. (A and B) Survival curves of *G. mellonella* infected with *C. glabrata* ATCC2001 at 7.5 × 10^5^ (squares), 1 × 10^6^ (triangles), 2.5 × 10^6^ (upside-down triangles), 5 × 10^6^ (diamonds) and 7.5 × 10^6^ (circles) cells/larva at 37°C (A) and 30°C (B). At 37°C (A) all infective doses displayed significant differences (p < 0.05), whereas at 30°C (B) all doses displayed significant differences (p < 0.05) except between the 2 lowest infective doses (7.5 × 10^5^ and 1 × 10^6^ cells/larva). (C) Larvae were infected with *C. glabrata* ATCC2001 at 1 × 10^6^, 2.5 × 10^6^, and 5 × 10^6^ cells/larva and at 2 and 6 hours post infection hemolymph was collected from larvae and OD_405_ determined to quantify melanin production. Larvae inoculated with PBS were used as a control, and asterisks denote a statistically significant difference to the PBS control (p < 0.05). (D) *G. mellonella* were infected with *C. glabrata* ATCC2001 at 2.5 × 10^6^ cells/larva and fungal burdens determined at the time points indicated. Scatterplots depict combined results from 3 independent replicates using 9 larvae for each time point

To evaluate *G. mellonella* as a host model for *C. glabrata* infection we first infected larvae with the commonly used wild type reference strain ATCC2001^28^ at a range of different inoculum levels (7.5 × 10^5^, 1 × 10^6^, 2.5 × 10^6^, 5 × 10^6^ and 7.5 × 10^6^ cells/larva). For this, groups of 20 healthy larvae (0.25–0.35 g) were inoculated with 10 µl of cell suspension through injection into the haemocoel with a Hamilton syringe through the last left pro-leg. Following infection larvae were incubated in the dark at 37°C and survival, based on response to physical stimulation, was monitored daily for 7 d. Larvae inoculated with PBS were used as uninfected controls and resulted in no deaths (data not shown), and all assays were performed at least 3 times independently. The results from this clearly demonstrated that *C. glabrata* can kill the larvae in a dose dependent fashion (Fig. 1A). For example, infection with 2.5 × 10^6^ cells/larva gave a mean survival time of 3.45 ± 0.28 d compared with 1.63 ± 0.13 d with an infective dose of 7.5 × 10^6^ cells/larva (P < 0.0001). The infective dose required for *C. glabrata* to kill *G. mellonella* larvae was however approximately 10-fold higher than the dose of *C. albicans* required to cause death, where an infective dose of 2 × 10^5^ cells/larva *C. albicans* NGY152^29^ cells resulted in a mean survival time of 2.95 ± 0.21 d (data not shown) similar to previous reports.^17^^,^^18^^,^^22^ Similar findings have been seen with other *Candida* species,^20-22^ and for *C. glabrata* is perhaps in keeping with its differing virulence properties favoring stealth and evasion over aggressive invasion.^23^^,^^30^ Given the high dose of *C. glabrata* required to cause *G. mellonella* killing we also evaluated the survival of larvae inoculated with heat-killed yeast cells (incubated at 75°C for 20 min before use), to ensure that killing was not due to other factors such as toxic shock. No larval death was seen following infection with heat-killed cells at 5 × 10^6^ cells/larva (data not shown); therefore the killing of larvae in this model is dependent on viable *C. glabrata* cells.

Temperature has been seen to impact on the outcome of *G. mellonella* infection for some fungal species, with both *C. albicans* and *C. tropicalis* displaying faster killing following incubation at 37°C rather than 30°C whereas killing rates following *C. krusei* infection were not affected by temperature.^21^^,^^22^^,^^31^ To determine if temperature also impacts on the virulence of *C. glabrata* we compared the virulence of *C. glabrata* ATCC2001 at 30 and 37°C. At the intermediate infective doses of both 1 × 10^6^ and 2.5 × 10^6^ cells/larva *C. glabrata* virulence was significantly decreased at 30°C compared with 37°C (Fig. 1B, P < 0.005). For example, with the 2.5 × 10^6^ cells/larva dose the mean survival time at 30°C was 4.65 ± 0.33 d compared with 3.45 ± 0.28 d at 37°C. However, no significant impact of temperature was seen when higher infective doses were used, suggesting this limitation can be overcome. The increased mortality seen at higher temperatures with intermediate infective doses may be the result of the impact of temperature on both the growth and virulence properties of the pathogen, plus the effect of temperature on the immune response of the larvae.^32^

Following infection with *C. glabrata* larvae quickly developed a brown-black colouration, indicative of the accumulation of melanin as part of the insect innate immune response. To quantify this hemolymph was collected from infected larvae, at 2 and 6 hours post infection, following established methods.^33^ The extent of melanisation was then determined through measuring the hemolymph optical density at 405 nm, which correlates with its visual appearance and has been used previously to quantify laccase activity.^22^ Through this the extent of melanisation was seen to be dependent on both the infective dose of *C. glabrata* and time post infection (Fig 1C). In addition to the activation of melanisation, previous work has demonstrated that the density of free hemocytes in the hemolymph is decreased following infection with *C. albicans* and other microbes.^21^^,^^22^^,^^24^ This decrease was seen to correlate with susceptibility to infection, and is thought to be the result of nodulation or clumping of hemocytes, pathogens and melanised debris at the infection site.^19^ Following infection with *C. glabrata*, at 2.5 × 10^6^ cells/larva, we also saw a significant decrease in total hemocyte cell density, with levels falling from 8.5 × 10^6^ ± 2.0 × 10^6^ cells/ml for PBS inoculated control larvae to 4.6 × 10^6^ ± 1.2 × 10^6^ (54%; p < 0.01) and 2.7 × 10^6^ ± 9.7 × 10^5^ (33%; p < 0.005) at 2 and 6 hours post infection respectively. Overall therefore, with the activation of melanisation and the drop in free hemocyte density, it is clear that the larvae mount a defense response following *C. glabrata* infection.

To follow the progress of infection we also determined the fungal burdens in *G. mellonella*, at 0, 6, 24, 48 and 72 h post infection, following inoculation with 2.5 × 10^6^ cells/larva *C. glabrata* ATCC2001. For this 9 larvae were taken at each time point, briefly washed in 70% ethanol followed by sterile water, and then placed into 15 ml screw-cap tubes with 4 stainless steel balls (3 mm) and 1 ml PBS. The tissue was then homogenized through 3 rounds of shaking for 20 s at 4 m/s in a Fastprep-24 (MP Biomedicals). The resulting homogenate was then suspended in 14 ml PBS and serial dilutions prepared and inoculated onto YEPD-chloramphenicol (100 µg/ml) plates. Immediately following infection the detectable fungal burden was 5.8 × 10^5^ ± 3.4 × 10^5^ CFU/larvae, and initially remained fairly constant only reaching 8.5 × 10^5^ ± 6 × 10^5^ CFU/larvae at 6 h post infection. However, by 24 h, when larvae began to succumb to infection, fungal burdens had risen ∼20-fold to 1.2 × 10^7^ ± 9.4 × 10^6^ CFU/larvae and continued to rise at 48 and 72 h post infection (Fig. 1D), demonstrating growth of the *C. glabrata* in the host.

**Figure 2.**
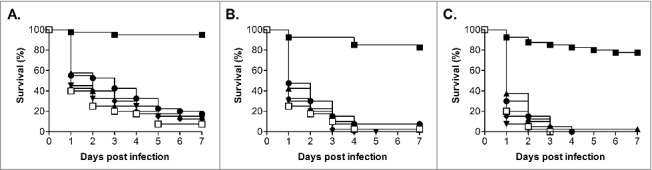
Virulence of *C. glabrata* clinical isolates in *G. mellonella*. Survival curves of *G. mellonella* infected with *C. glabrata* ATCC2001 (open squares), NCPF3605 (closed squares), BG2 (triangles), Cg1184 (upside-down triangles), Cg85/038 (diamonds), and Cg11088A (circles) at 1 × 10^6^ (A), 2.5 × 10^6^ (B), and 5 × 10^6^ (C) cells/larva. At all doses strain NCPF3605 was highly attenuated compared with all other strains (p < 0.0001); plus at the highest dose (C) a slight but significant increase in virulence was seen for Cg1184 compared with BG2 (p < 0.005) and Cg11088A (p < 0.05)

In addition to testing the ability of *C. glabrata* ATCC2001 to cause lethal infection in *G. mellonella* we also screened a further 5 clinical isolates (BG2, Cg1184, Cg85/038, Cg11088A and NCPF3605),^34-37^ including the other commonly used isolate BG2, at 3 infective doses (1.25 × 10^6^, 2.5 × 10^6^ and 5 × 10^6^ cells/larva; Fig. 2). All strains demonstrated a dose dependent response, and 4 of these strains (BG2, Cg1184, Cg85/038 and Cg11088A) demonstrated a very similar level of virulence to ATCC2001. Indeed when comparing the different infective doses the only significant difference was the slight increase in virulence seen with strain Cg1184 at the 5 × 10^6^ cells/larva dose compared with BG2 (P < 0.005) and Cg11088A (P < 0.05). Therefore the majority of *C. glabrata* isolates tested show broadly similar levels of virulence in this model. The only key exception was the NCPF3605 strain which, at all infective doses tested, was clearly highly attenuated in virulence compared with all the other isolates (P < 0.0001) only causing 22.5% ± 9.6% killing of larvae by 7 d at the highest dose tested. A recent comparison of the growth of this strain and ATCC2001, at varying glucose concentrations, demonstrated distinct differences, with NCPF3605 displaying a faster growth rate but at the cost of entering stationary phase at a lower cell density.^38^ General fitness defects may therefore impact on the virulence of *C. glabrata* in this model and, importantly, the *G. mellonella* model can be used to detect differences in the virulence potential of *C. glabrata* strains.

Auxotrophic markers are commonly used for selection during the genetic modification of *Candida* species. However, *in C. albicans* it is well recognized that *ura3* auxotrophy causes virulence to be highly attenuated in the mouse model of infection, and that the level of expression of *URA3* can also impact on virulence.^29^^,^^39^ In this work we have demonstrated that *ura3* auxotrophy has a similar impact on the virulence of *C. glabrata* in the *G. mellonella* infection model. *G. mellonella* infected with 2. 5 × 10^6^ cells/larva of wild type *C. glabrata* (ATCC2001) demonstrated a mean survival time of 2.90 ± 0.19 d compared with 6.39 ± 0.13 d (p < 0.0001) for those infected with a *ura3* auxotroph (strain 2001U^40^). Given the known issues with *ura3* auxotrophy in *C. albicans*, most work in *C. glabrata* uses other auxotrophic markers that have been shown not to impact on virulence in mice.^23^^,^^41^ We therefore also screened *his3, leu2* and *trp1* single mutants (strains 2001H, 2001L and 2001T respectively) and a *his3, leu2, trp1* triple mutant (strain 2001HTL).^41^ Similar to the situation in a mouse model, loss of *HIS3* or *LEU2* did not impact on microbial virulence within the *G. mellonella* model (data not shown). However, in our model we did detect a subtle, but significant, attenuation of virulence in the *trp1* single (mean survival time 3.67 ± 0.20 days, p = 0.0173) and *his3, leu2, trp1* triple null mutant (mean survival time 3.87 ± 0.20 days, p = 0.0020) compared with the wild type strain ATCC2001 (mean survival time of 2.90 ± 0.19 days). It is tempting to attribute the lower virulence of the triple mutant to its loss of *trp1*; however, as we did not test this empirically we cannot rule out the possibility that the combined auxotrophies also impact on fitness and ultimately virulence. Overall, we would therefore suggest that when using this model it is important to ensure appropriate control strains, displaying the same auxotrophies, are used.

To assess the utility of this model to screen defined mutants for virulence defects we also performed virulence assays with 14 deletion mutants, including 8 whose phenotype in a mouse model of infection has previously been reported. These included 12 mutants associated with *C. glabrata* stress responses (Δ*ste50*, Δ*ste20*, Δ*hog1*, Δ*skn7*, Δ*yap1*, Δ*cta1*, Δ*rim101*, Δ*yps1*, Δ*msn2*, Δ*msn4*, Δ*cst6* and Δ*slt2*)^41-43^ plus 2 glycosylation mutants (Δ*mnn2* and Δ*anp1*)^44^ that have previously been shown to be hypervirulent in mice. To increase the statistical power of the assay we used 50 larvae per replicate, and selected one infective dose (2.5 × 10^6^ cells/larva) which would allow either an increase or decrease in virulence potential to be observed. Virulence was then compared with the relevant wild type strain (2001HTL, 2001H or HT6^41,43^), selected based on the genetic background of the mutant. Through this analysis we identified 5 mutants that demonstrated a mild but significant attenuation in virulence, including Δ*ste50*, Δ*ste20*, Δ*hog1* in the Hog pathway, Δ*slt2* in the cell wall integrity pathway and Δ*skn7* involved in oxidative stress resistance (Table 1). The level of attenuation in these mutants was generally subtle, but shown to be significant through the benefit of being able to infect a large number of larvae to increase the statistical power of the assay. Of these mutants 4, Δ*ste20*, Δ*hog1*, Δ*slt2* and Δ*skn7*, have previously been tested in a mouse model of systemic infection where they also displayed a mild (Δ*ste20*, Δ*slt2* and Δ*skn7*) to moderate (Δ*hog1*) attenuation of virulence.^45-48^ For *STE50* this is the first report of its importance in virulence, and the phenotype displayed by the Δ*ste50* mutant is in keeping with the overall importance of the Hog1 pathway. Furthermore, the large scale analysis of *C. glabrata* mutants in the *Drosophila* infection model^13^ also identified both the Hog1 and cell wall integrity pathways as playing a key role in virulence, therefore the importance of these pathways has been consistently demonstrated in 3 different infection models. The remaining mutants (Δ*yap1*, Δ*cta1*, Δ*rim101*, Δ*yps1*, Δ*msn2*, Δ*msn4* and Δ*cst6*; Table 1) displayed no significant defect in virulence, and of these 2 (Δ*yap1* and Δ*cta1*) have also previously been reported to display no virulence defect in mice.^49^^,^^50^ In addition to the stress response mutants we also screened 2 glycosylation mutants (Δ*mnn2* and Δ*anp1*)^44^ that have previously been shown to demonstrate increased virulence in a mouse model of infection. Intriguingly, these deletion mutants also displayed increased virulence in the *G. mellonella* model while their complemented strains demonstrated wild type virulence (Table 1). The molecular basis of this hypervirulence is currently not clear, but the mutants are known to be hyperadherent and potentially elicit a septic-shock like response.^44^ This may therefore suggest that either similar components are involved in the recognition of the pathogen by *G. mellonella* or common adhesins play a role in both models. Overall, of the 14 mutants tested in this study 8 have previously been screened in mice and we have shown all to display comparable phenotypes in the *G. mellonella* model, plus confirm the importance of the Hog1 and cell wall integrity pathways in virulence. This correlation is very encouraging and suggests that this model has the potential to be used to screen for novel virulence factors in *C. glabrata.*

**Table 1.** Virulence of *C. glabrata* mutants in the *G. mellonella* model

Strain	Mean Survival Time (days)	Log rank test(P value)	LT_50_ (days)	L.V.I.
Wild type (2001HTL)^41^	2.41 ± 0.12	−	1.37	−
Δ*hog1*^41^	3.30 ± 0.41	<0.005	2.31	−0.52
Δ*skn7*^41^	3.87 ± 0.25	<0.0001	3.30	−0.88
Δ*rim101*^41^	2.27 ± 0.09	N.S.	1.34	0.02
Δ*yps1*^41^	2.87 ± 0.11	N.S.	1.84	−0.30
Wild type (2001HTL)^41^	2.32 ± 0.12	−	1.29	−
Δ*ste50*^41^	3.99 ± 0.10	<0.0001	3.22	−0.92
Δ*slt2*^41^	3.26 ± 0.23	<0.0005	2.26	−0.56
Δ*ste20*^41^	3.09 ± 0.19	<0.01	2.04	−0.46
Δ*cta1*^41^	2.41 ± 0.16	N.S.	1.45	−0.12
Wild type (HT6)^43^	2.44 ± 0.19	−	1.42	−
Δ*yap1**^42^*	2.57 ± 0.42	N.S.	1.48	−0.04
Wild type (2001H)^41^	3.32 ± 0.37	−	2.30	−
Δ*msn4*^41^	3.40 ± 0.25	N.S.	2.41	−0.05
Δ*cst6*^41^	3.01 ± 0.15	N.S.	2.06	0.11
Δ*msn2*^41^	2.93 ± 0.20	N.S.	1.92	0.18
Wild type (HT6)^43^	3.17 ± 0.28	−	2.20	−
Δ*mnn2**^44^*	2.11 ± 0.16	<0.0001	1.22	0.59
Δ*mnn2+MNN2**^44^*	3.29 ± 0.48	N.S.	2.30	−0.05
Wild type (HT6)^43^	2.79 ± 0.09	−	1.71	−
Δ*anp1**^44^*	1.54 ± 0.16	<0.0001	0.73	0.85
Δ*anp1+ANP1**^44^*	2.64 ± 0.13	N.S.	1.57	0.09

*Note.* (L.V.I. Larval virulence index, N.S. Not significant).

To facilitate the future use of this model for comparing mutant strains, potentially through large scale screening efforts, we also calculated a larval virulence index (LVI) as a measure of virulence for the set of 14 mutants tested. For this we followed the methodology established for use with the *Drosophila* infection model.^13^^,^^41^ Survival curves were initially fitted to a Weibull distribution, then the time of 50% larval survival (LT_50_) determined and from this the LVI presented as the log_2_ ratio of mutant and corresponding wild type control (Table 1). The LT_50_ values determined for the different strains were, as expected, in very strong agreement with their mean survival times (Spearman's rho = 0.99, P < 0.00001). Furthermore, applying the cut-offs established by Brunke *et al*.^13^ for increased or decreased virulence (virulence index ± 0.5), 6 of the 7 mutants we identified as displaying altered virulence through the traditional log rank tests were also highlighted by this approach. The mutant not highlighted, Δ*ste20*, was on the verge of detection with a LVI of −0.46 and was also the least attenuated in virulence through the traditional log rank test. Overall, therefore, this modeling approach gives a strong quantitative measure of virulence, and may facilitate the future use of this model in the large scale screening of available *C. glabrata* deletion libraries.^41^

**Figure 3.**
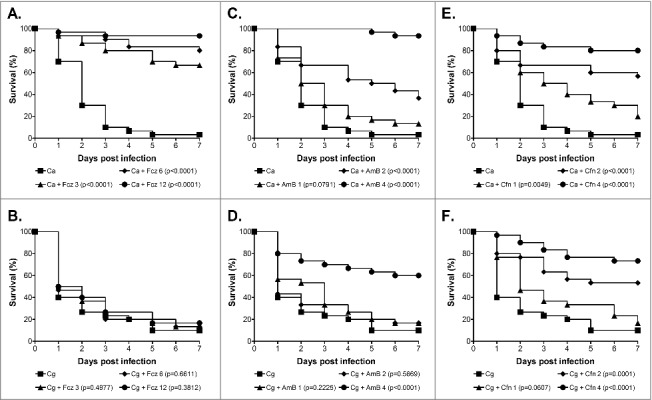
Antifungal efficacy against *C. albicans* and *C. glabrata* in the *G. mellonella* model. Survival curves of *G. mellonella* infected with 1 × 10^5^ cells/larva *C. albicans* NGY152 (A, C and E), or 1.25 × 10^6^ cells/larva *C. glabrata* ATCC2001 (B, D and F). (A and B) Fluconazole treatment at 0 (squares), 3 (triangle), 6 (diamonds) or 12 mg/kg (circles). (C, D, E and F) Amphotericin B or Caspofungin treatment at 0 (squares), 1 (triangle), 2 (diamonds) or 4 mg/kg (circles)

Finally, we also tested the efficacy of fluconazole, amphotericin B, and caspofungin against *C. glabrata* in the *G. mellonella* model and compared this to *C. albicans. C. glabrata* is well recognized for displaying a higher innate tolerance to azole antifungals than *C. albicans.*^10^^,^^11^ Consistent with this, although *C. albicans* NGY152 was highly sensitive to fluconazole *in vitro* the *C. glabrata* strain ATCC2001 displayed a high MIC of 32 µg/ml, whereas for caspofungin and amphotericin B both were acutely sensitive. We next tested the efficacy of these antifungals in the *G. mellonella* model at clinically relevant doses that did not cause toxicity in the model (data not shown). For this, larvae were infected with 1 × 10^5^ cells/larva *C. albicans* NGY152 or 1.25 × 10^6^ cells/larva *C. glabrata* ATCC2001 and antifungals administered 30 min post infection through a second 10 µl injection into the pro-leg adjacent to the site of initial infection. Untreated controls received a second injection of PBS. For *G. mellonella* infected with *C. albicans,* treatment with fluconazole at all concentrations tested (3, 6, and 12 mg/kg) promoted survival (Fig 3A; P < 0.0001). In contrast treatment with the same levels of fluconazole provided no protection against *C. glabrata* infection (Fig. 3B). Amphotericin B at both 2 and 4 mg/kg also protected larvae against infection by *C. albicans* (Fig. 3C; P < 0.0001), whereas only the highest concentration (4 mg/kg) provided significant protection against *C. glabrata* (Fig. 3D; P < 0.0001). Finally, caspofungin also protected *G. mellonella* against *C. albicans* infection at all concentrations tested (Fig. 3E, 1 mg/kg P < 0.005; 2 and 4 mg/kg P < 0.0001), whereas again only the higher doses of 2 and 4 mg/kg provided significant protection against *C. glabrata* infection (Fig. 3F, P < 0.0001). We therefore saw a clear correlation between *in vitro* susceptibility and *in vivo* efficacy in this model for *C. glabrata*, consistent with previous studies demonstrating the potential of the *G. mellonella* model to be used in testing the toxicity and efficacy of antifungal agents for a range of fungal pathogens.^21^^,^^22^^,^^31^^,^^51^^,^^52^ Interestingly, although amphotericin B and caspofungin did provide protection against *C. glabrata,* in both cases a higher dose of antifungal was required to achieve efficacy than for treatment of *C. albicans*. A similar profile was seen following infection with *C. krusei* and this was associated with the strain tested, although being susceptible, demonstrating reduced susceptibility compared with the *C. albicans* control strain.^22^ In this work however, the *C. glabrata* and *C. albicans* strains used demonstrated very similar susceptibility profiles toward amphotericin B and caspofungin. The basis of this subtle but consistent *in vivo* susceptibility shift is therefore not clear. It could simply be due to the requirement of a tenfold higher infective dose for *C. glabrata* compared with *C. albicans*, which may result in the need for a higher drug concentration, or alternatively it could be suggestive of *C. glabrata* displaying a higher *in vivo* tolerance toward antifungals.

Infection models using *G. mellonella* are generally gaining acceptance and have now been established for a range of fungal pathogens. As previously discussed these models present some advantages through being more ethically acceptable, inexpensive allowing the use of more test subjects to increase the statistical power of the assay, alongside the easy manipulation of larvae and ability to assay at 37°C. There are however some disadvantages such as no complete genome sequence and the lack of genetic tractability in *G. mellonella*, plus an inherent level of variability in the quality of larvae from suppliers. Finally, as with any infection model, it is unlikely that all virulence attributes involved in mammalian infection will demonstrate similar importance in the *G. mellonella* system. Overall however we would conclude that *G. mellonella* is an attractive and simple model for following *C. glabrata* infection. High doses are initially required to cause an infection, but once established a simple to follow lethal infection coupled with growth of the pathogen and a detectable host response is seen. Furthermore, through the mutants tested, we saw a good level of correlation with murine models suggesting that this system has the potential to be used to screen for novel virulence factors in this important pathogen. Finally, as has been seen with other fungal pathogens, this system can clearly be used for the *in vivo* evaluation of antifungal agents.
